# Birth Defects Surveillance in the United States: Challenges and Implications of International Classification of Diseases, Tenth Revision, Clinical Modification Implementation

**DOI:** 10.1155/2014/212874

**Published:** 2014-10-29

**Authors:** Adel Mburia-Mwalili, Wei Yang

**Affiliations:** ^1^Environmental Sciences and Health Graduate Program, University of Nevada, Reno, NV 89557, USA; ^2^School of Community Health Sciences, University of Nevada, Reno, NV 89557, USA

## Abstract

Major birth defects are an important public health issue because they are the leading cause of infant mortality. The most common birth defects are congenital heart defects, neural tube defects, and Down syndrome. Birth defects surveillance guides policy development and provides data for prevalence estimates, epidemiologic research, planning, and prevention. Several factors influence birth defects surveillance in the United States of America (USA). These include case ascertainment methods, pregnancy outcomes, and nomenclature used for coding birth defects. In 2015, the nomenclature used by most birth defects surveillance programs in USA will change from ICD-9-CM to ICD-10-CM. This change will have implications on birth defects surveillance, prevalence estimates, and tracking birth defects trends.

## 1. Introduction

Birth defects are an important public health issue because they are the leading cause of infant mortality in the United States of America (USA) causing one in every five infant deaths [1]. In USA, birth defects affect about 3% of births [2]. Worldwide, birth defects are the fourth leading cause of neonatal deaths [3]. An estimated 7.9 million children (6% of births) are born with a major birth defect every year globally [4]. In 2010, about 9% of all neonatal deaths in 193 countries around the world were due to birth defects [3]. Morbidity and mortality among children with birth defects are high and the health care costs are enormous. In 2004, billed costs for hospitalizations for birth defects in USA were estimated to be 2.6 billion dollars [5].

The most common birth defects are congenital heart defects [3, 6, 7], neural tube defects, and Down syndrome [3]. Several studies on the etiology of birth defects suggest that multiple factors cause some birth defects [8, 9]. These factors include genetics [10], environmental factors [8, 9], and gene-environment interactions [11]. Despite several decades of birth defects research, the causes of nearly half of all birth defects are still unknown [3].

Birth defects surveillance provides data for prevalence estimates, epidemiologic research, planning, and prevention and it guides policy development [12]. However, birth defects surveillance faces several challenges that make it complex to estimate national and international prevalence. These include case ascertainment methods, pregnancy outcomes, and nomenclature used by various birth defects surveillance programs.

This paper reviews the challenges of birth defects surveillance in USA and explores the implications of the implementation of the International Classification of Diseases, Tenth Revision, Clinical Modification (ICD-10-CM) in the year 2015 and its potential impact on birth defects surveillance.

## 2. Birth Defects Surveillance

Public health surveillance is the systematic and continuous collection, management, analysis, and interpretation of data which is disseminated in a timely manner to individuals working in public health [13]. Interest in birth defects surveillance was sparked by the thalidomide tragedy of the 1960s when an increased number of children with limb deformities were born in Germany and other parts of the world where thalidomide was used for treating nausea and morning sickness among pregnant women [14].

Following the thalidomide tragedy, the Metropolitan Atlanta Congenital Defects Program (MACDP), the first population-based birth defects surveillance program in USA, was established by Centers for Disease Control and Prevention (CDC) in 1967 to conduct birth defects surveillance [15]. The Birth Defects Prevention Act of 1998 helped accelerate the establishment of birth defects surveillance programs in other states. Presently, most states have an established birth defects surveillance program, even though a few states are yet to implement such a program [16].

The National Birth Defects Prevention Network (NBDPN), a volunteer-based organization which works in collaboration with CDC, was established in 1997. NBDPN's goals are to maintain a national network of state- and population-based birth defects surveillance programs and to be involved in birth defects research and prevention. The NBDPN has done a tremendous job of improving the uniformity of birth defects surveillance in USA and also provides technical assistance to states whenever needed. In 2004, NBDPN published guidelines for conducting birth defects surveillance [17].

In 2013, there were 43 population-based birth defects surveillance programs in USA and 41 of these programs reported data on select birth defects to NBDPN [16, 18]. This is almost two-thirds the number of programs that reported select birth defects data to NBDPN in 2000 [12]. CDC's National Center on Birth Defects and Developmental Disabilities funds 14 of these state-based birth defects surveillance programs. In addition, CDC also funds the Centers for Birth Defects Research and Prevention which is involved in large birth defects studies, such as the multistate National Birth Defects Prevention Study (NBDPS) conducted from 1997 to 2013. The Birth Defects Study to Evaluate Pregnancy Outcomes, a multistate birth defect study, will build on NBDPS. It started data collection in January 2014.

Global birth defects surveillance and research are conducted by the International Clearinghouse for Birth Defects Surveillance and Research (ICBDSR), a voluntary nonprofit organization affiliated with World Health Organization (WHO) that was established in 1974 [19]. Currently, there are 45 birth defects surveillance programs worldwide that are members of ICBDSR, and membership is by program and not country. Countries with more than one birth defect surveillance program can have several programs as members of ICBDSR. The majority of the member programs are from Europe (15 countries), Asia (5 countries), North America (3 countries), South America (3 countries), and Australia (2 countries) [20]. USA has six birth defects surveillance programs that are members of ICBDSR. These include Arkansas, Atlanta, California, Iowa, Texas, and Utah. African countries are yet to have birth defects surveillance programs join ICBDSR.

### 2.1. Case Ascertainment

Physical examination of infants provides the best assessment of birth defects; however, this is very expensive and most birth defects surveillance programs cannot afford this resource intensive method. Conversely, birth defects are underreported in birth certificates; therefore this method of surveillance does not capture all cases of birth defects in a given population [17]. Consequently, states use various case ascertainment methods for birth defects surveillance in order to capture all potential birth defects cases in their population of interest. The method used by each state depends on the program's purpose, the state's birth defects legislation, resources available, collaboration with the community, and partners involved in birth defects surveillance [16].

Birth defects data can be obtained from multiple sources which provide the necessary information for each case. Birth and pediatric hospitals can be used to obtain a majority of the cases. However, other data sources, such as laboratories and outpatient clinics, may provide birth defects data. Case ascertainment methods used by birth defects programs include active, passive, and active-passive (hybrid).

Active case ascertainment is the preferred method for birth defects surveillance because program staff go out to find birth defects cases from hospitals, clinics, and other health care facilities [17]. This method is resource intensive but provides the most accurate information in a timely manner [21]. In addition, programs that use active surveillance generally provide the highest birth defects prevalence estimates because they are more comprehensive in capturing all possible birth defects cases [12]. Often, birth defects that are ascertained actively are usually confirmed and not probable [22].

In passive case ascertainment, the birth defects surveillance program receives reports of cases of infants with birth defects from different data sources such as hospitals, clinics, and other sources. These sources may be voluntary or are mandated by law [14]. The completeness and accuracy of data may be varied for programs that use this method because the quality of data is dependent on the data source [17]. This method is inexpensive because program staff do not have to make contacts with hospitals or other birth defects reporting sources. However, since different institutions report data, data quality and timeliness may be an issue [21]. In addition, since verification of reported cases is not done, some of the birth defects may be probable and not confirmed [22].

Some birth defects surveillance programs use active-passive case ascertainment, a hybrid approach, whereby birth defects cases are reported to them by hospitals and other reporting facilities just as in passive surveillance. However, program staff uses various methods to ascertain the cases from these sources [17]. For example, a certain percentage of all reported birth defects cases or some specific birth defects can be actively ascertained [23]. This method improves the data quality because false positive cases can be easily identified.

In 2013, 43 population-based birth defects surveillance programs reported data to NBDPN; of these, 17 (40%), 13 (30%), and 13 (30%) used active case finding, passive case finding, and passive case finding with active case ascertainment, respectively [24].

Data from the three case ascertainment methods may be comparable; however, birth defects surveillance programs that use passive case ascertainment need to incorporate various measures to ensure that the birth defects reports they receive are an accurate representation of birth defects in their targeted population [17]. This may be achieved by linking data for reported cases to hospital discharge data to capture infants with birth defects discharged from hospitals. Hospital discharge data has been shown to be a valuable source of birth defects cases even though it does not identify all infants with birth defects [22, 25]. It may be difficult to capture infants born at home, especially if they do not seek medical care or if they seek medical care outside the birth defects program catchment area or in another state. It would be ideal for states to have data sharing agreements for birth defects surveillance such that, irrespective of where a child with a birth defect seeks treatment, the information will be passed on to the child's resident state's birth defects surveillance program. Most states already have data sharing agreements for cancer and new birth cases and the same idea could easily be done for birth defects surveillance. However, it is unclear how many states or birth defects surveillance programs have data sharing agreements in place. NBDPN mainly facilitates most of the multistate collaborative birth defects research projects.

Researchers linked data for select birth defects from two independent birth defects surveillance programs in Florida that used active and passive case ascertainment methods, respectively [22]. The geographic area for the two surveillance systems overlapped, and the goal was to evaluate the sensitivity and completeness of the active and passive case ascertainment. They reported that the ability of the passive birth defects surveillance was limited and dependent on the birth defects codes. For example, the ability to identify cases of anencephaly was a challenge because most infants with the defect are stillborn or die shortly after birth; thus the hospital rarely created a record for such a case. In addition, they reported that the enhanced system that used case ascertainment was able to rule out false positives in the passive surveillance after medical records review. Thus, passive surveillance had a reduced positive value.

Another study used 2006–2010 nationwide Down syndrome data reported to NBDPN by 41 population-based birth defects surveillance programs [16]. The prevalence estimates ranged from 10.2 to 20.0 per 10,000 births and 6.9 to 20.6 per 10,000 births for programs that used active and passive case ascertainments methods, respectively (Table 1).

### 2.2. Pregnancy Outcomes

Population-based birth defects surveillance programs include live births only, live births and stillbirths only, and live births, stillbirths, and elective terminations (all pregnancy outcomes) in their birth defects case definition. The pregnancy outcome included by a birth defects surveillance program depends on the purpose, resources available, and access to the pregnancy outcome information [17]. Birth defects programs that include all pregnancy outcomes provide the most accurate prevalence estimates. Of the 41 birth defects surveillance programs in USA that reported data to NBDPN in 2013, 29% reported data from live births only, 42% reported data from live births and stillbirths, and 29% reported data from all pregnancy outcomes [16].

Ethen and Canfield compared birth defects prevalence rates for elective terminations of any gestation and elective terminations of at least 20-week gestation or 500-gram birth weight [26]. They reported an increase of 5% or more for the following birth defects: anencephaly, spina bifida without anencephaly, encephalocele, Patau syndrome (trisomy 13), Edwards syndrome (trisomy 18), Down syndrome (trisomy 21), omphalocele, gastroschisis, and anophthalmia. A recent study using birth defects data from MACDP reported Down syndrome prevalence of 16.3 per 10,000 live births among all pregnancy outcomes and 11.5 per 10,000 lives among live births only [27]. In addition, another study also using birth defects data from MACDP reported a prevalence of Patau syndrome (trisomy 13) of 0.63 per 10,000 live births among live births only and 1.57 per 10,000 live births among all pregnancy outcomes [28]. Furthermore, they also reported a prevalence of Edwards syndrome (trisomy 18) of 1.16 per 10,000 live births among live births only and 4.01 per 10,000 live births among all pregnancy outcomes. Researchers using data from 41 population-based surveillance programs that reported data to NBDPN in 2013 found a higher prevalence of Down syndrome when all pregnancy outcomes were included compared to prevalence estimates from live births only [16] (Table 2).

It is imperative that prevalence estimates that use data from live births only are interpreted cautiously because the above studies clearly demonstrate that including all pregnancy outcomes provides the most accurate birth defects prevalence estimates. In addition, elective terminations should include cases of any gestation and not be limited to those equal to or greater than 20-week gestation. Moreover, there is need for a general consensus on whether elective terminations will include all cases irrespective of gestation age and birth weight or include elective termination of at least 20-week gestation and birth weight of 500 grams [12, 26]. This may be a challenge for some birth defects surveillance programs because including stillbirths and elective terminations may involve active case ascertainment which is resource intensive and it engages more partners in birth defects surveillance, such as clinics that conduct elective terminations. Additionally, the added dimension of including all birth outcomes in the birth defects surveillance may not align with the purpose of some birth defects surveillance programs.

Medical advances have made it possible for prenatal screening and detection of birth defects during pregnancy. Most prenatal procedures occur in outpatient settings and active surveillance would be the best method for prenatally diagnosed birth defects because of the followup needed with the outpatient clinics to abstract cases of birth defects. Birth defects surveillance programs that use passive surveillance and rely on hospitals and other reporting facilities would be faced with the challenge of receiving reports of prenatally diagnosed birth defects. Cragan and Gilboa conducted a study using data from outpatient prenatal diagnostic clinics to estimate birth defects prevalence [29]. They noted an increase in the prevalence of specific birth defects even though the increase in prevalence of all birth defects was small. They also reported that the prenatal diagnosis records had birth defects categorized as definite or possible which posed a challenge on whether to include either definite or possible cases in the prevalence estimates. They reported separate prevalence estimates with and without possible cases. Prenatally diagnosed birth defects in population-based birth defects surveillance are an evolving field where guidelines are needed.

### 2.3. Nomenclature/Disease Classification Systems

The International Classification of Diseases (ICD) is the standard method for coding morbidity and mortality data to monitor disease incidence, prevalence, and other health conditions [30]. ICD-10 is the most current version used worldwide, except in USA, and it was implemented by the majority of WHO member states in 1994. WHO has scheduled to release ICD-11 in 2017. In USA, the ICD-9-CM, a modified version of ICD-9, was implemented in 1979 after WHO implemented the ICD-9 in 1975. ICD-10-CM, a modified USA version of ICD-10, is scheduled to be implemented in USA in October 2015 [31].

#### 2.3.1. International Classification of Diseases, Tenth Revision, Clinical Modification (ICD-10-CM)

In 1990, WHO published ICD-10 in a continued effort for detailed descriptions of diseases and health conditions in the ever changing medical field, with new diseases and health conditions being added frequently. Most countries around the world use this coding scheme for both morbidity and mortality [30]. However, in USA, ICD-10 has been used for mortality coding only since 1999. Nonetheless, USA has been working on the ICD-10-CM, the USA clinical modification of the ICD-10, which is comparable to ICD-10, for morbidity coding. ICD-10-CM was initially scheduled to be implemented in October 2013, but this implementation date was rescheduled for 2014. Unfortunately, the implementation date was rescheduled again for October 2015 [31]. The ICD-10-CM has over 60,000 alphanumeric diagnoses codes which use five to seven characters that allow more specific reporting of diseases and new health conditions. In addition, the sixth digit captures clinical details and the added codes now show laterality [31]. ICD-10-CM birth defects codes range from Q00 to Q99. ICD-10-CM is much improved compared to ICD-9-CM. For example, in ICD-9-CM coding scheme, a single code, 756.79, was assigned for both omphalocele and gastroschisis. But now, ICD-10-CM has two distinct codes: omphalocele (Q79.2) and gastroschisis (Q79.3).

NBDPN has already translated ICD-9-CM birth defects codes to ICD-10-CM (Table 3). However, caution must be exercised because back translation of ICD-10-CM to ICD-9-CM cannot be done since there are many more ICD-10-CM birth defects codes that may not be translated to ICD-9-CM. NBDPN has developed a separate guidance on how to translate ICD-10-CM back to ICD-9-CM. The transition to ICD-10-CM while being very beneficial and long overdue may pose some challenges for birth defects surveillance. For instance, nine months of data for calendar year 2015 will be coded using ICD-9-CM codes and three months of data for the same calendar year will be coded in ICD-10-CM. Additionally, it may be misleading to compare some birth defects prevalence data before and after the ICD-10-CM transition because of increased specificity of the ICD-10-CM coding scheme.

Some shortcomings of ICD-10-CM include a lack of distinction between birth defects among premature and mature infants such as patent ductus arteriosus. In addition, polydactyly, although not a major birth defect, does not have a code to indicate the position of the extra digit [32].

Researchers used Alberta Congenital Anomalies Surveillance System (ACASS) data to compare an adaptation of the ICD-10-Royal College of Paediatrics and Child Health (ICD-10-RCPCH) coding with ICD-9-British Paediatric Association (ICD-9-BPA) [33]. ACASS transitioned from ICD-9-BPA to ICD-10-RCPCH in 2000. It was found that some birth defects codes in ICD-10-RCPCH had moved to different sections or organ systems; there were more individual and detailed codes for congenital syndromes, and it required more detailed codes or less detailed codes for some anomalies such that ACASS had to create their own codes for tetralogy of Fallot for more specificity. Moreover, the registry noted a significant difference for congenital hip dislocation prevalence estimates using ICD-10 coding because ICD-10 has more codes compared to ICD-9. Besides, ACASS has continued to use both ICD-10-RCPCH and ICD-9-BPA for data requests because some birth defects cannot be collapsed into one major group. For instance, tetralogy of Fallot can be easily collapsed into one group using ICD-9-BPA. However, this is not the case in ICD-10-RCPCH because one of the defects that make up tetralogy of Fallot has been moved to another grouping of heart defects.

Moczygemba and Fenton conducted a pilot study to evaluate the use of ICD-10-CM for diabetes, heart disease, and pneumonia [34]. The researchers found several validity-type errors such as incorrect assignment of the seventh-character extension, failure to use placeholders, and incomplete ICD-10-CM codes. It was concluded that although the ICD-10-CM is more robust, the increased specificity of health conditions may be challenging to find the specific code needed and that there is a varying degree of proficiency among coders depending on education level, clinical background, and training which may lead to inconsistent code assignment.

The full impact of the implementation of ICD-10-CM will be best evaluated after all the healthcare facilities transition to the new system. However, birth defects surveillance should be aware of some of the anticipated issues and address them accordingly. In addition, WHO will release the ICD-11 in 2017, two years after the proposed implementation of ICD-10-CM in USA. It is questionable whether USA will be ready to implement the ICD-11 soon after its release in order to allow comparison of morbidity and mortality data with the rest of the world.

#### 2.3.2. International Classification of Diseases, Ninth Revision, Clinical Modification (ICD-9-CM)

ICD-9-CM is the coding scheme that has been used in USA for over 30 years to code diagnoses and procedures during a hospital encounter. It is also used for research, hospitalization rates, and estimation of healthcare costs. ICD-9-CM is based on 1978 WHO's ICD-9 and was modified to meet statistical needs in USA and implemented in 1979 [35]. The ICD-9-CM includes more than 13,000 diagnoses codes and uses more digits in the codes than WHO's ICD-9; thus diseases are described more specifically [36]. It uses three to five numeric codes and at least two codes are needed to code etiology and manifestation. The ICD-9-CM is updated every October in order to be current with the ever changing medical field. However, as much as the ICD-9-CM was intended to be more accurate, the coding scheme has outlived its usefulness and does not adequately capture all the current medical conditions, resulting in inaccuracies in reporting health conditions. ICD-9-CM birth defects codes range from 740.0 to 759.9.

Over half of the 43 USA population-based birth defects surveillance programs that report data to NBDPN use ICD-9-CM to code birth defects [24]. This has had an impact on birth defects surveillance in USA because the coding scheme has not kept up with the changes in the medical field. For instance, gastroschisis and omphalocele both have the same ICD-9-CM code of 756.79 and yet they are distinct birth defects. In 2009, NBDPN introduced separate codes for these two birth defects in order to make a distinction [32].

#### 2.3.3. Centers for Disease Control and Prevention/British Paediatric Association Classification

The British Paediatric Association (BPA) modified the ICD-9 in 1979 to be used for pediatric and neonatal cases. The codes range from 740.000 to 759.999 in order to be similar to ICD-9 codes. The first four digits match the ICD-9; however, the fifth digit is specific to children. In 1983, the staff of CDC's Division of Birth Defects and Developmental Disabilities, National Center on Birth Defects and Developmental Disabilities, modified the BPA coding system and created a 6-digit code for the birth defects classification system to provide more details of a birth defect [17]. In addition, the sixth digits “5,” “6,” and “7” are used, for instance, when a more detailed description is needed, which cannot be captured by the first five digits [37]. For example, more specificity for spina bifida is demonstrated in the following codes:

 741.085 spina bifida, meningocele, cervicothoracic, with hydrocephalus,
 741.086 spina bifida, meningocele thoracolumbar, with hydrocephalus,
 741.087 spina bifida, meningocele, lumbosacral, with hydrocephalus [17].

The CDC/BPA coding scheme while being very detailed has some shortcomings. These include being complicated to use; comprehensive coding instructions for some birth defects are not provided; it is over 30 years old and the birth defects field has evolved in this time period making some codes outdated, and individuals using the coding scheme need to be familiar with medical terminology, human anatomy, and birth defects [37].

Like the ICD-10-CM coding system, the 6-digit CDC-modified BPA system allows a more robust system that provides greater specificity of birth defects, laterality of the defect, whether a defect is possible or probable or diagnosed only prenatally, and related conditions. For example, omphalocele is coded as 756.700 and gastroschisis is coded as 756.710. In addition, the CDC/BPA coding scheme is even more detailed than the ICD-10-CM for some birth defects such as spina bifida without anencephalus in which CDC/BPA has 21 codes compared to 10 and 2 codes for ICD-10-CM and ICD-9-CM, respectively.

## 3. Future Directions

Birth defects surveillance in USA faces some challenges and a more standardized surveillance method used by all birth defects programs is needed. Cancer surveillance and the behavioral risk factor surveillance system (BRFSS) core questions have standardized surveillance procedures. Furthermore, the aforementioned surveillance systems are federally funded in all 50 states, District of Columbia (DC), and USA territories. Yet, only one-third of state-based birth defects surveillance programs receive federal funding [38]. With reduced funding in most public health programs, federal funding for birth defects surveillance in all 50 states may not be feasible soon. In addition, some states do not even have a birth defects surveillance system yet, although it is 16 years after the Birth Defects Prevention Act of 1998. With the implementation of ICD-10-CM in 2015, it may be misleading to compare birth defects prevalence estimates using ICD-10-CM and ICD-9-CM because in general ICD-10-CM has more codes. However, for some birth defects such as congenital cataract, ICD-9-CM has more codes than ICD-10-CM and CDC/BPA coding schemes, respectively. Additionally, NBDPN is yet to translate CDC/BPA to ICD-10-CM even though some birth defects surveillance programs are ready for the implementation of ICD-10-CM [24]. It may be beneficial for birth defects surveillance to apply partly the BRFSS model. BRFSS is a state-based telephone health survey that collects health-related risk behaviors, chronic health conditions, and use of preventive services data yearly from all 50 states, DC, Puerto Rico, the USA Virgin Islands, Guam, American Samoa, and Palau among noninstitutionalized adults aged 18 years and over. BRFSS has three sets of questions: the core (fixed) questionnaire which is asked every year by all states, the rotating core which has questions that are asked every other year, optional modules, and the state-added questions which give states the autonomy to ask questions that are specific to each state's individual needs [39]. In 2011, BRFSS methodology changed to include cell phones and the weighting methodology changed. Therefore, data from years prior to 2011 may not be comparable to data after the methodological change.

BRFSS and birth defects surveillance are inherently very different; however, some guidelines from BRFSS such as having core, rotating, optional, and state-added modules or questions may be applicable to birth defects surveillance. Categorization of birth defects reported to NBDPN, for instance, core and optional, may be very useful especially after October 2015 once the ICD-10-CM is implemented. NBDPN recently revised the birth defects list which will be implemented soon by birth defects surveillance programs. The revised birth defects list has now categorized birth defects as core, recommended, and extended (Cara Mai, MPH, e-mail communication, July 14, 2014). The revision of the birth defects list will potentially increase reporting of all core birth defects to NBDPN by most birth defects surveillance programs. Currently, NBDPN has a list of 45 birth defects [40] that are not categorized and it may be daunting for some programs that have limited resources to report all or some of the 45 birth defects on the list (Table 3). The revision of the NBDPN birth defects list is very timely, especially after the ICD-10-CM is implemented in 2015. Birth defects surveillance programs will still be at liberty to use other coding schemes such as the CDC/BPA if they so wish and will also be able to track other birth defects that may be of interest to them. This approach will ensure a standard coding scheme of reporting core birth defects and will allow comparison across all birth defects surveillance programs in USA. Of course the issues of case ascertainment methods and pregnancy outcomes included by birth defects surveillance programs would persist, but at least the nomenclature used by birth defects surveillance programs would be uniform.

## 4. Conclusion

Birth defects surveillance programs in USA use various case ascertainment methods (passive versus active surveillance), include various pregnancy outcomes (live births only, live births and stillbirths, and all pregnancy outcomes), and use different nomenclature (ICD-9-CM and CDC/BPA) in their surveillance efforts. The change in nomenclature from ICD-9-CM to the more comprehensive ICD-10-CM in 2015 will have an impact on birth defects surveillance, especially the comparison of data in the two coding systems. Individual state's birth defects surveillance legislation and resources available greatly determine the scope of birth defects surveillance efforts of state programs. However, the effects of this nomenclature change can only be fully assessed once the implementation of ICD-10-CM has occurred.

## Figures and Tables

**Table 1:** Down syndrome prevalence from population-based birth defects surveillance programs by case ascertainment methods, United States, 2006–2010 [16].

Case ascertainment method	Prevalence per 10,000 births
Active case finding (*n* = 15)	10.2–20.0
Passive case finding^*^ (*n* = 26)	6.9–20.6

^*^With or without case ascertainment.

**Table 2:** Down syndrome prevalence from population-based surveillance programs by pregnancy outcome, United States 2006–2010 [16].

Pregnancy outcome	Prevalence per 10,000 births
Live births (*n* = 12)	6.9–15.6
Live births and stillbirths (*n* = 17)	8.8–17.0
All pregnancy outcomes (*n* = 12)	10.2–20.6

**Table 3:** Birth defects list with ICD-9-CM, ICD-10-CM, and CDC/BPA codes, National Birth Defects Prevention Network [41].

Birth defects	ICD-9-CM codes	CDC/BPA codes	ICD-10-CM codes
Central nervous system			
Anencephalus	740.0-740.1	740.00–740.10	Q00.0-Q00.1
Spina bifida without anencephalus	741.0–741.9 without 740.0–740.10	741.00–741.99 without 740.0–740.10	Q05.0–Q05.9, Q07.01, Q07.03 without Q00.0-Q00.1
Hydrocephalus without spina bifida	742.3 without 741.0, 741.9	742.30–742.39 without 741.00–741.99	Q03.0–Q03.9
Encephalocele	742.0	742.00–742.09	Q01.0–Q01.9
Microcephalus	742.1	742.10	Q02
Eye			
Anophthalmia/microphthalmia	743.0, 743.1	743.00–743.10	Q11.0–Q11.2
Congenital cataract	743.30–743.34	743.32	Q12.0
Aniridia	743.45	743.42	Q13.1
Ear			
Anotia/microtia	744.01, 744.23	744.01, 744.21	Q16.0, Q16.1
Cardiovascular			
Common truncus	745.0	745.00	Q20.0
Transposition of great arteries	745.10, 745.11, 745.12, 745.19	745.10–745.19 (exclude 745.13, 745.15, and 745.18)	Q20.1, Q20.3, Q20.5
Tetralogy of Fallot	745.2	745.20-745.21, 747.31	Q21.3
Ventricular septal defect	745.4	745.40–745.49 (exclude 745.487, 745.498)	Q21.0
Atrial septal defect	745.5	745.51–745.59	Q21.1
Atrioventricular septal defect (endocardial cushion defect)	745.60, 745.61, 745.69	745.60–745.69, 745.487	Q21.2
Pulmonary valve atresia and stenosis	746.01, 746.02	746.00-746.01	Q22.0, Q22.1
Tricuspid valve atresia and stenosis	746.1	746.10 (exclude 746.105)	Q22.4
Ebstein's anomaly	746.2	746.20	Q22.5
Aortic valve stenosis	746.3	746.30	Q23.0
Hypoplastic left heart syndrome	746.7	746.70	Q23.4
Patent ductus arteriosus	747.0	747.00	Q25.0
Coarctation of aorta	747.10	747.10–747.19	Q25.1
Total anomalous pulmonary venous return (TAPVR)	747.41	747.42	Q26.2
Orofacial			
Cleft palate without cleft lip	749.0	749.00–749.09	Q35.0–Q35.9
Cleft lip with and without cleft palate	749.1, 749.2	749.10–749.29	Q36.0–Q36.9, Q37.0–Q37.9
Choanal atresia	748.0	748.0	Q30.0
Gastrointestinal			
Esophageal atresia/tracheoesophageal fistula	750.3	750.30–750.35	Q39.0–Q39.4
Rectal and large intestinal atresia/stenosis	751.2	751.20–751.24	Q42.0–Q42.9
Pyloric stenosis	750.5	750.51	Q40.0
Hirschsprung's disease (congenital megacolon)	751.3	751.30–751.34	Q43.1
Biliary atresia	751.61	751.65	Q44.2-Q44.3
Genitourinary			
Renal agenesis/hypoplasia	753.0	753.00-753.01	Q60.0–Q60.6
Bladder exstrophy	753.5	753.50	Q64.10, Q64.19
Obstructive genitourinary defect	753.2, 753.6	753.20–753.29 and 753.60–753.69	Q62.0–Q62.3 and Q64.2-Q64.3
Hypospadias	752.61	752.60–752.62 (exclude 752.61 and 752.621)	Q54.0–Q54.9 (exclude Q54.4)
Epispadias	752.62	752.61	Q64.0
Musculoskeletal			
Reduction deformity, upper limbs	755.20–755.29	755.20–755.29	Q71.0–Q71.9
Reduction deformity, lower limbs	755.30–755.39	755.30–755.39	Q72.0–Q72.9
Gastroschisis^*^	756.79	756.71	Q79.3
Omphalocele^**^	756.79	756.70	Q79.2
Congenital hip dislocation	754.30, 754.31, 754.35	754.30	Q65.0–Q65.2
Diaphragmatic hernia	756.6	756.61	Q79.0-Q79.1
Chromosomal			
Patau syndrome (trisomy 13)	758.1	758.10–758.19	Q91.4–Q91.7
Down syndrome (trisomy 21)	758.0	758.00–758.09	Q90.0–Q90.9
Edwards syndrome (trisomy 18)	758.2	758.20–758.29	Q91.0–Q91.3
Other			
Fetus or newborn affected by maternal alcohol use	760.71	760.71	Q86.0
Amniotic bands	No code	658.80	No code

*Note.*

ICD-9-CM: International Classification of Diseases, Ninth Edition, Clinical Modification.

ICD-10-CM: International Classification of Diseases, Tenth Edition, Clinical Modification.

CDC/BPA codes: Centers of Disease Control and Prevention/British Paediatric Association.

^*^756.79 started being coded as 756.73 as of 10/1/2009.

^**^756.79 started being coded as 756.72 as of 10/1/2009.
